# Biological Distribution and Metabolic Profiles of Carbon-11 and Fluorine-18 Tracers of VX- and Sarin-Analogs in Sprague–Dawley Rats

**DOI:** 10.1021/acs.chemrestox.0c00237

**Published:** 2020-12-29

**Authors:** Thomas R. Hayes, Chih-Kai Chao, Joseph E. Blecha, Tony L. Huynh, Kurt R. Zinn, Charles M. Thompson, John M. Gerdes, Henry F. VanBrocklin

**Affiliations:** † Department of Radiology and Biomedical Imaging, University of California at San Francisco, San Francisco, California 94143, United States; ‡ Department of Biomedical and Pharmaceutical Sciences, University of Montana, Missoula, Montana 59812, United States; § Departments of Radiology, Small Animal Clinical Sciences, and Biomedical Engineering; Institute for Quantitative Health Science and Engineering, Michigan State University, East Lansing, Michigan 48824, United States

## Abstract

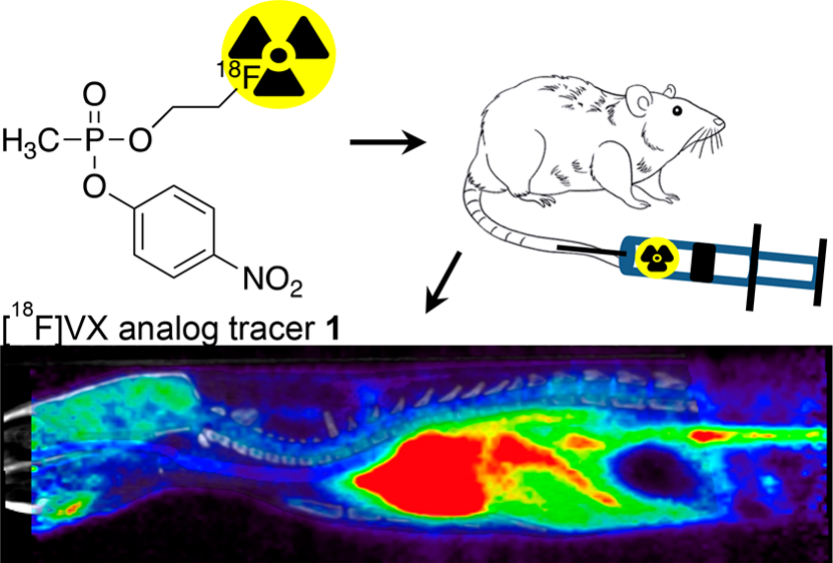

Organophosphorus esters (OPs) were originally developed as pesticides but were repurposed as easily manufactured, inexpensive, and highly toxic chemical warfare agents. Acute OP toxicity is primarily due to inhibition of acetylcholinesterase (AChE), an enzyme in the central and peripheral nervous system. OP inhibition of AChE can be reversed using oxime reactivators but many show poor CNS penetration, indicating a need for new clinically viable reactivators. However, challenges exist on how to best measure restored AChE activity in vivo and assess the reactivating agent efficacy. This work reports the development of molecular imaging tools using radiolabeled OP analog tracers that are less toxic to handle in the laboratory, yet inhibit AChE in a similar fashion to the actual OPs. Carbon-11 and fluorine-18 radiolabeled analog tracers of VX and sarin OP agents were prepared. Following intravenous injection in normal Sprague–Dawley rats (*n* = 3–4/tracer), the tracers were evaluated and compared using noninvasive microPET/CT imaging, biodistribution assay, and arterial blood analyses. All showed rapid uptake and stable retention in brain, heart, liver, and kidney tissues determined by imaging and biodistribution. Lung uptake of the sarin analog tracers was elevated, 2-fold and 4-fold higher uptake at 5 and 30 min, respectively, compared to that for the VX analog tracers. All tracers rapidly bound to red blood cells (RBC) and blood proteins as measured in the biodistribution and arterial blood samples. Analysis of the plasma soluble activity (nonprotein/cell bound activity) showed only 1–6% parent tracer and 88–95% of the activity in the combined solid fractions (RBC and protein bound) as early as 0.5 min post injection. Multivariate analysis of tracer production yield, molar activity, brain uptake, brain area under the curve over 0–15 min, and the amount of parent tracer in the plasma at 5 min revealed the [^18^F]VX analog tracer had the most favorable values for each metric. This tracer was considered the more optimal tracer relative to the other tracers studied and suitable for future in vivo OP exposure and reactivation studies.

## Introduction

Organophosphorus esters (OPs) are a class of compounds which were originally created as pesticides; however, due to their human toxicity, they were subsequently developed into chemical warfare agents (CWAs). CWAs such as the V- (e.g., VX) and G- (e.g., sarin, soman) series are nondiscriminating poisons and constitute a threat to both military and civilian populations. The primary mechanism of action of OP agents is via the inhibition of acetylcholinesterase (AChE), the enzyme responsible for the hydrolysis of the neurotransmitter acetylcholine (ACh).^1^ Exposure to high concentrations of OPs results in a rapid increase of synaptic ACh and triggers neurotoxic sequelae through the depletion of AChE in both the central and peripheral nervous systems.^1−6^

**Scheme 1:**
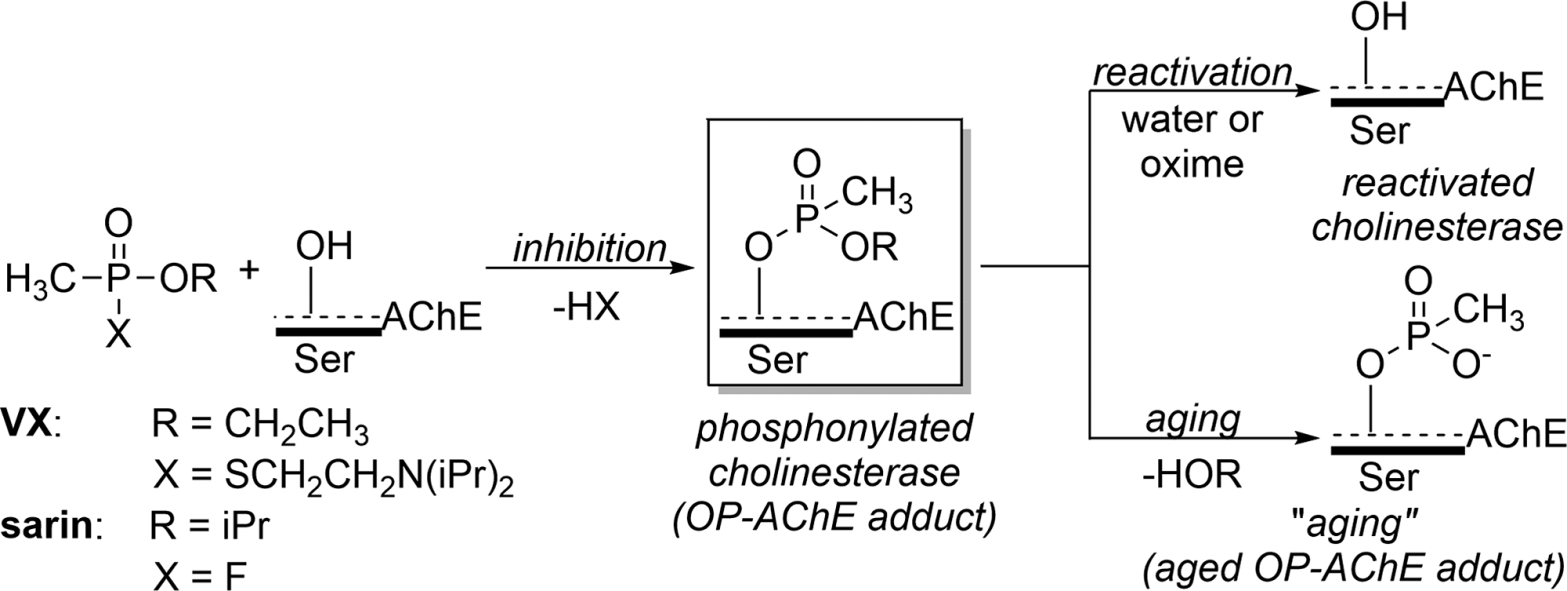
Reaction of OP CWA’s with Acetylcholinesterase and Subsequent Recovery or Aging Processes

Inhibition of AChE by OP agents is mediated through the formation of a covalent bond with the active site serine in the enzyme (Scheme 1).^7^ Primary standard of care (SOC) pharmacotherapy for OP exposures include oximes, anticholinergic drugs (atropine), and an anticonvulsant (diazepam). Oximes represent a potential antidote for OP poisoning, with 2-pralidoxime chloride (2-PAM) being the current SOC. While inhibition of AChE may be reversed using oxime reactivators, many show poor CNS penetration, creating a need for new clinically viable reactivators.^8^ Reactivation of AChE by oximes involves displacement of the OP moiety from the active site through nucleophilic attack on the adducted phosphorus atom of the enzyme.^2,9,10^ In some cases, permanent deactivation of the enzyme occurs through an “aging” process, resulting in the formation of a methyl phosphonate anion conjugate with the serine residue, which is refractory to reactivation.^11,12^ Inhibition of AChE by the V- and G-series CWAs all undergo aging; however, the aging rate is only minutes in the case of soman, whereas sarin and VX age far more slowly, 5 and 24 h, respectively.^5,13^ Due to their continued threat to both military personnel and civilians, development of more effective treatments is necessary to further safeguard against the misuse of OPs.

Precise diagnostic tools are needed to assess the interaction of the OPs with AChE in vivo and also to provide a means to assess antidote intervention. One method that can be used to directly assess in vivo OP pharmacokinetics and AChE interaction is molecular imaging with positron emission tomography (PET). Previously, analogs of VX and sarin were developed with a 4-nitrophenol (PNP) moiety in place of either the fluorine (sarin) or thiol (VX) leaving groups.^14^ These OP analogs were shown to form identical adducts with AChE relative to VX and sarin CWAs but with decreased volatility and toxicity.^14,15^ Incorporation of a radioisotope on the alkoxyl moiety of the OP yields a compound which can be visualized and quantified in vivo as the AChE-OP adduct. By definition the replacement of a carbon-12 with carbon-11 would produce a radiolabeled surrogate of VX and sarin when bound to AChE. The addition of a fluorine to the side chain phosphodiester altered the resultant reactivity (reactivation/aging) of an OP-AChE adduct; however, fluorine substitution did not dramatically alter inhibitory potency because the electronegative effect was sufficiently insulated from the electrophilic reaction site (phosphorus atom).^16,17^ The longer half-life of the fluorine-18 labeled compounds, analogs of VX and Sarin, enabled tissue assessments. Although the term surrogate has been used in the past, in this publication all of the radioactive OP compounds will be called VX and Sarin analogs.

As using a live agent is restricted in the U.S., the PNP leaving group (nerve agent analogs) has been shown to lead to identical OP-AChE adducts. Changing to a PNP leaving group does lower the reactivity slightly but the analogs remain potent phosphorylating agents and like paraoxon and parathion, binding to albumin is expected to be low.^18^ Synthetic methods for PET analogs of both VX and sarin with carbon-11 (half-life = 20 m) and fluorine-18 (half-life = 110 m) have been reported;^19−22^ however, only the [^18^F]VX analog tracer in vivo analysis has been presented.^21^ In this study, we compared the distribution and metabolism of four PET OP analog radiotracers (Table 1), [^18^F]VX analog, **1**, [^18^F]sarin analog, **2**, [^11^C]VX analog, **3**, and [^11^C]sarin analog, **4**, in rats using both in vivo and ex vivo methods in order to select the most optimal tracer for future in vivo studies.

**Table 1:**
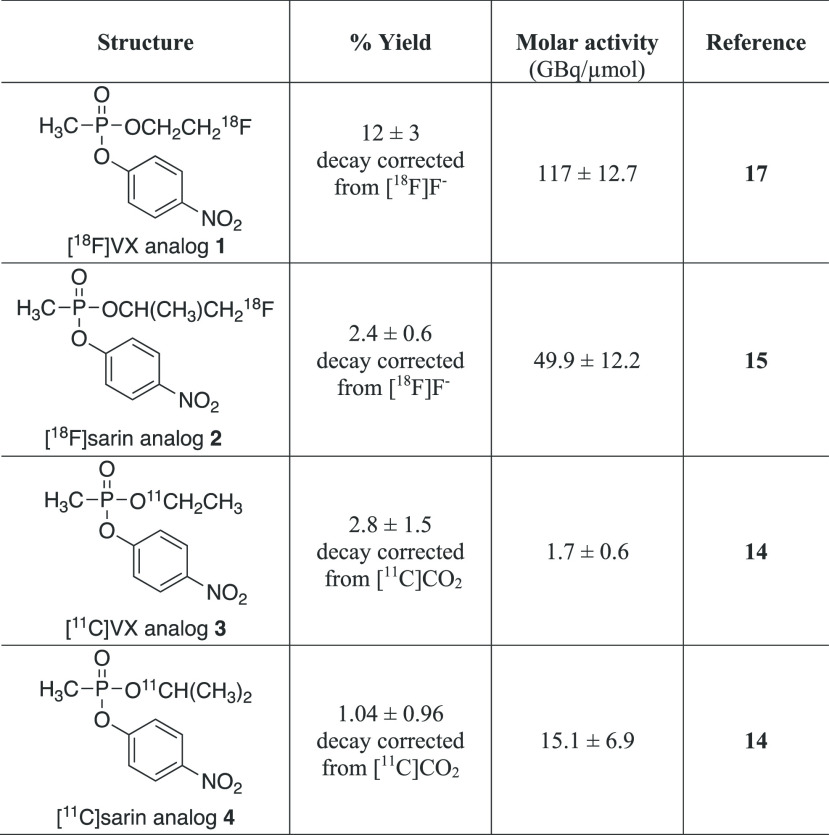
Tracer Structure, Radiochemical Yield, and Molar Activity of OP Analogs Studied

a Decay corrected (d.c.).

## Experimental Procedures

Chemicals were received from Sigma-Aldrich or Fisher Scientific and used without further purification. Radioisotopes were obtained using a GE PETtrace medical cyclotron through either a ^18^O(p,n)^18^F reaction on [^18^O]H_2_O enriched water or ^14^N(p,α)^11^C reaction on [^14^N]N_2_ with 1% O_2_. *O*-(2-[^18^F]Fluoroethyl), *O*-(4-nitrophenyl) methylphosphonate, [^18^F]VX analog **1,***O*-(1-[^18^F]fluoropropan-2-yl), *O*-(4-nitrophenyl) methylphosphonate, [^18^F]sarin analog **2**, 1-[^11^C]ethyl (4-nitrophenyl)methylphosphonate, [^11^C]VX analog **3**, 2-[^11^C]propyl (4-nitrophenyl)methylphosphonate, and [^11^C]sarin analog **4**, were synthesized as previously reported in comparable yields and molar activities (Table 1) and formulated in 10% CH_3_CN/10 mM phosphate buffer saline (PBS) pH 6.8.^19,20,22^ HPLC analysis of arterial blood samples was performed on a Waters 590 system coupled to a Shimadzu SPD UV–visible detector (Columbia, MO) Hamilton PRP-1 5 μm, 250 × 4.6 mm column with a 50:50 CH_3_CN:phosphate buffer (5 mM; pH 6.8) isocratic eluent at 1 mL/min. Tracer stability studies were performed at 1 h via analytical HPLC using a Luna C-18(2) column (Phenomenex, C-18(2) 5 μ, 250 × 4.6 mm) with a 40:60 pH 6.8 phosphate buffer (5 mM): CH_3_CN mobile phase at 1.5 mL/min. Counting of radioactive samples was performed on a HIDEX automatic gamma counter using the included software. Counts were normalized by diluting a known activity of tracer to 100 mL with H_2_O, and 1 mL was sampled 5 times to determine response of the counter. Biodistribution and arterial samples were decay corrected to time of injection.

### Sprague–Dawley Rats

Wild-type male Sprague–Dawley rats (250–450 g) were purchased from Charles Rivers Laboratory (Wilmington, MA) and acclimated for 1 week after receipt prior to imaging and biodistribution studies. Studies were conducted at the University of California, San Francisco (UCSF), accredited by the American Association for Accreditation of Laboratory Animal Care (AAALAC). The rat experiments were performed under a UCSF IACUC approved protocol, adhering to all NIH requirements and institutional regulations. All rats were communally housed, at least 2 rats per cage, with environmental enrichment. Rats were fed a nutritionally complete rat chow ad libitum with free access to water prior to the studies.

### Biodistribution

Sprague–Dawley rats were anesthetized with isoflurane (1–1.5%) and were maintained under anesthesia with warming and constant monitoring throughout the study period until euthanasia. Each tracer was administered individually by intravenous (i.v.) tail vein injection (0.37–1.48 MBq/animal, *n* = 3). Blood (1–3 mL) was drawn by cardiac puncture at 5 or 30 min from the anesthetized rats followed by cervical dislocation and death confirmed by pneumothorax. Brain, liver, heart, kidney, lung (^11^C and ^18^F tracers), and bone (^18^F tracers only) were harvested. The tissues were placed on absorbent paper, and specifically, the heart was squeezed, to drain the excess blood prior to weighing and counting. An energy-calibrated HIDEX automatic gamma counter (HIDEX Oy, Turku, Finland) was used to obtain decay corrected (time of injection) activity and weight for each of the tissues. Percent injected dose per gram of tissue (% ID/g) was calculated using a known activity standard.

### Rat microPET/CT

A dedicated small animal PET/CT (Inveon, Siemens Medical Solutions, Malvern, PA) was used for all imaging procedures. All doses (0.3–1 mL, 25–52 MBq) were administered as i.v. bolus injections via a tail vein catheter, followed by a 0.3 mL saline flush. Three to four rats were imaged per tracer. The PET and CT imaging was performed normothermic (37 °C) under anesthesia (isoflurane 2–2.5%) with warming and constant monitoring. Dynamic PET imaging data were acquired over 60 min (tracers **3** and **4**) or 90 min (tracers **1** and **2**) beginning at the time of tracer injection.

PET data reconstruction was performed using vendor-provided software. An iterative reconstruction algorithm with CT-based attenuation correction was used for PET, and a Feldkamp reconstruction algorithm modified for conebeam was used for CT. The reconstructed volumes were 128 × 128 × 159 matrices with a voxel size of 0.776383 mm × 0.776383 mm × 0.796 mm for PET and 512 × 512 × 700 matrices with an isotropic voxel size of 0.196797 mm × 0.196797 mm × 0.196797 mm for CT. The CT acquisition parameters were continuous 120 rotation steps over 220×, 80 kVp/500 μA tube voltage/current, and 175 ms exposure per step.

The reconstructed CT and PET imaging data were processed with AMIDE open source software version 1.0.5^23^ and oriented as defined by Paxinos and Watson.^24^ The X, Y, Z coordinates of imaging views were centered at bregma and the regions of interest (ROIs) were defined according to the rat anatomy. The PET scan tissue radioactivity values were reported as standardized uptake values (SUVs). A time-activity curve (TAC) was generated using the SUVs against the middle of the time frames. The area under the curve (AUC) was computed by the trapezoidal rule using R(Version 3.5.2).^25^ One-way ANOVA was performed using Microsoft Excel (version 16.16.12) or R (version 3.5.2).^25^ A *P*-value <0.05 was considered statistically significant.

### Arterial Blood Sampling for Metabolite Analysis

Arterial sampling was performed on animals during in vivo imaging. Before tracer injection, a tail ventral arterial catheter was installed. Arterial blood (∼0.2 mL) was drawn at 0.5, 1, 5, 10, 30, and 60 min after tracer injection. Prior to each blood collection the dead volume in the arterial catheter was removed and discarded. After each blood collection, and at 20 and 45 min, the arterial catheter was flushed with heparinized saline (0.3 mL). Approximately 100 μL of collected blood was then placed in a heparinized 1.5 mL centrifuge tube containing citric acid (25 μL, 10 mg/mL in H_2_O). The tubes were centrifuged at 13 200*g* for 1 min at room temperature to remove the red blood cells. The plasma fraction was removed and placed in a second tube with acetonitrile (100 μL), to precipitate the proteins, mixed by repeated inversion and centrifuged at 13 200*g* for 1 min. The plasma/CH_3_CN fraction was then removed from the protein fraction and placed in third centrifuge tube. The supernatant was diluted with 200 μL of deionized (DI) H_2_O and analyzed by analytical reversed-phase HPLC. The HPLC eluent was collected in 2 fractions, 0–5 min (polar metabolites) and 5–10 min (parent tracer). The red blood cells, protein, and separated HPLC eluents were then counted on a HIDEX automatic gamma counter and the percent in each fraction was calculated as the percentage of all recovered activity from each sample.

Stability of the tracers under the conditions used for processing the arterial samples was also assessed. A sample of 100 μL of formulated tracer was processed using the same conditions as the arterial blood samples. Decomposition of the tracer was then assessed by radio-HPLC using the same arterial sampling column and mobile phase.

## Results and Discussion

The radiolabeled OP analog tracers **1**–**4** (Table 1), fluorine-18 labeled VX and sarin analogs, and also carbon-11 labeled VX and sarin analogs, were prepared as previously described^19−22^ in which the radiosynthesis outcomes are defined in Table 1. Comparisons of the tracer production metrics, radiochemical yields, and molar activities are shown. In general the fluorine-18 analogs trended toward higher radiochemical yield than the corresponding carbon-11 analogs. The molar activities of the fluorine-18 tracers were greater than the carbon-11 tracers, which may be due to endogenous CO_2_ in the reactor and/or CO_2_ introduced when diluting the methyl Grignard reagent.^15^ The molar activities for all of the analog tracers were sufficient for the imaging assessment studies given the high capacity of acetylcholinesterase to react with the tracers in vivo.

The [^18^F]VX analog tracer was originally prepared by reaction of [^18^F]fluoroethyltosylate with the 4-nitrophenyl hydrogen methylphosphonate and formulated in 10% CH_3_CN/pH 7.4 phosphate buffered saline (PBS).^21^ As the synthetic approach to the sarin and VX analogs was refined through the common bis(4-nitrophenyl)methylphosphonate so too was the final formulation, 10% CH_3_CN/PBS pH 6.8.^19,20,22^ The analogs were found to decompose more rapidly in solutions at pH greater than 7 and exhibited improved solubility at pH less than 7. One of the known decomposition products was the hydrolysis of the PNP to give the P–OH. Stability studies performed 1 h post formulation showed that all four tracers had >99% radiochemical purity using the lower pH formulation.

The initial pharmacokinetic profiles (0–30 min post injection) and metabolic profiles of the radiolabeled analogs were evaluated in vivo in naïve rats. Given that the OP agents rapidly react with AChE after injection, the baseline in vivo behavior of these tracers at the early time points was thought to be more representative of this AChE mode of action and potentially key for the future assessments of antidote intervention. The analog tracers were administered at a very low dose for the imaging studies (∼0.06–7.35 μg per ∼0.3 kg rat), depending on the molar activity (Table 1) and the mCi administered. For the biodistribution studies, the administered dose was 18–140 fold lower than the imaging study doses. A mass dose of 30–75 μg of the OP would be needed to see a measurable decrease in AChE inhibition activity, so there was no pharmacological or toxicological consequence on AChE interaction at the administered imaging or biodistribution study doses.

**Figure 1:**
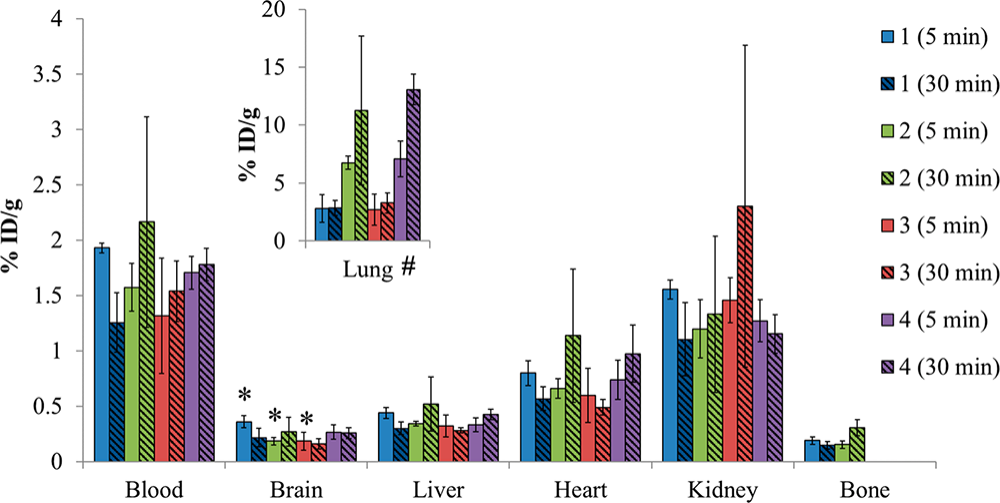
Tissue distribution of the four analogs at 5 min, solid, and 30 min, hashed, post injection. **1**: [^18^F]VX analog, blue; **2**: [^18^F]sarin analog, green; **3**: [^11^C]VX analog, red; **4**: [^11^C]sarin analog, purple. The percent injected dose per gram of tissue (%ID/g) values presented as mean ± SD (*n* = 3). * 5 min brain uptake of [^18^F]VX analog is significantly greater than 5 min [^11^C]VX analog and 5 min [^18^F]sarin analog uptake (*p* < 0.05). **#** [^18^F]sarin analog and [^11^C]sarin analog exhibited significantly greater uptake at 5 and 30 min in lung tissue versus [^18^F]VX and [^11^C]VX (*p* < 0.05).

The ex vivo biodistributions at 5 and 30 min post injection are compiled as shown in Figure 1. The [^18^F]VX analog **1** demonstrated significantly higher uptake in the brain at 5 min compared to [^18^F]sarin analog (**2**) and [^11^C]VX analog (**3**). For all the tracers, there was minimal washout of the brain activity over 30 min. Radioactivity uptake profiles in the liver, heart and kidney were similar across all the analogs at each time point. Additionally, minimal defluorination of the two fluorine-18 analog tracers was observed as evidenced by limited uptake of [^18^F]fluoride into bone. The most significant uptake and retention of activity was found in the lung (Figure 1; inset). The VX tracers exhibited an uptake of ∼3% (2.71–3.29) injected dose per gram of tissue at 5 and 30 min post injection, whereas the sarin analogs showed significant (*P* < 0.05) 2-fold higher uptake at 5 min and 4-fold higher uptake at 30 min. The concentrations of butyrylcholinesterase (BChE) and carboxyesterase (CarbE) are known to be relatively high in rat plasma and lung and, therefore upon reaction with these radiolabeled OPs, may account for the observed elevated values.^26,27^

**Figure 2:**
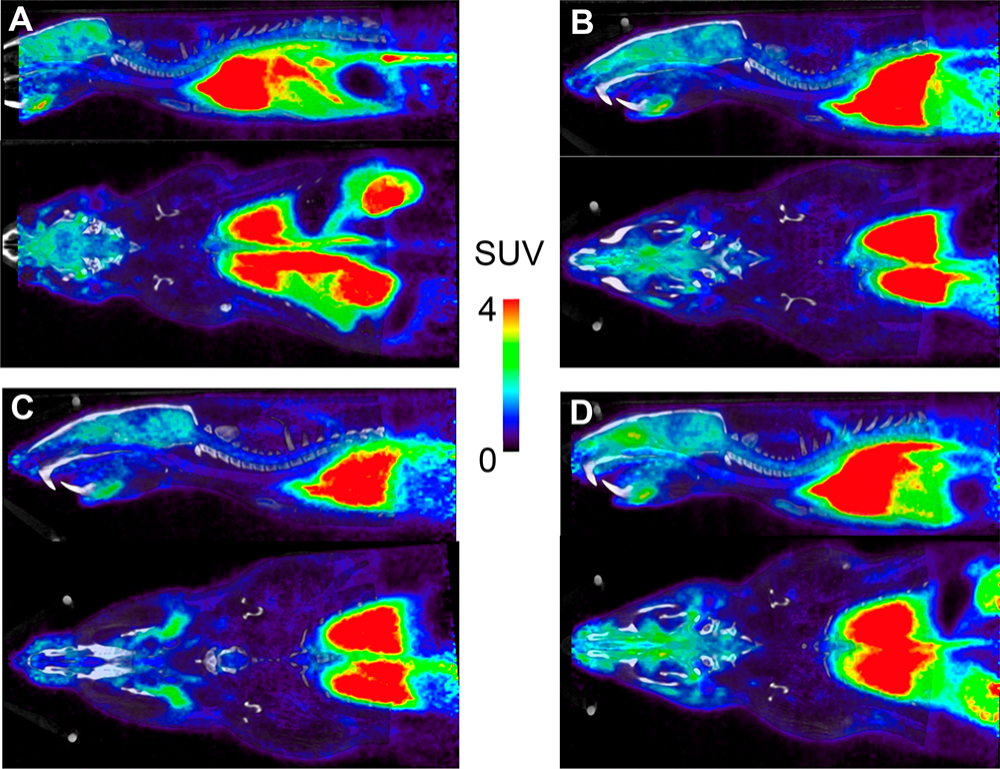
Two PET/CT views per panel as sagittal (upper) and transverse (lower) views of Sprague–Dawley rats (summed radioactivity, 0–15 min) as A: [^18^F]VX analog, **1**; B: [^18^F]sarin analog, **2**; C: [^11^C]VX analog, **3**; D: [^11^C]sarin analog, **4**, as **s**tandardized uptake values (SUV) heat maps correlated to the NIH color bar defined SUV range.

**Figure 3:**
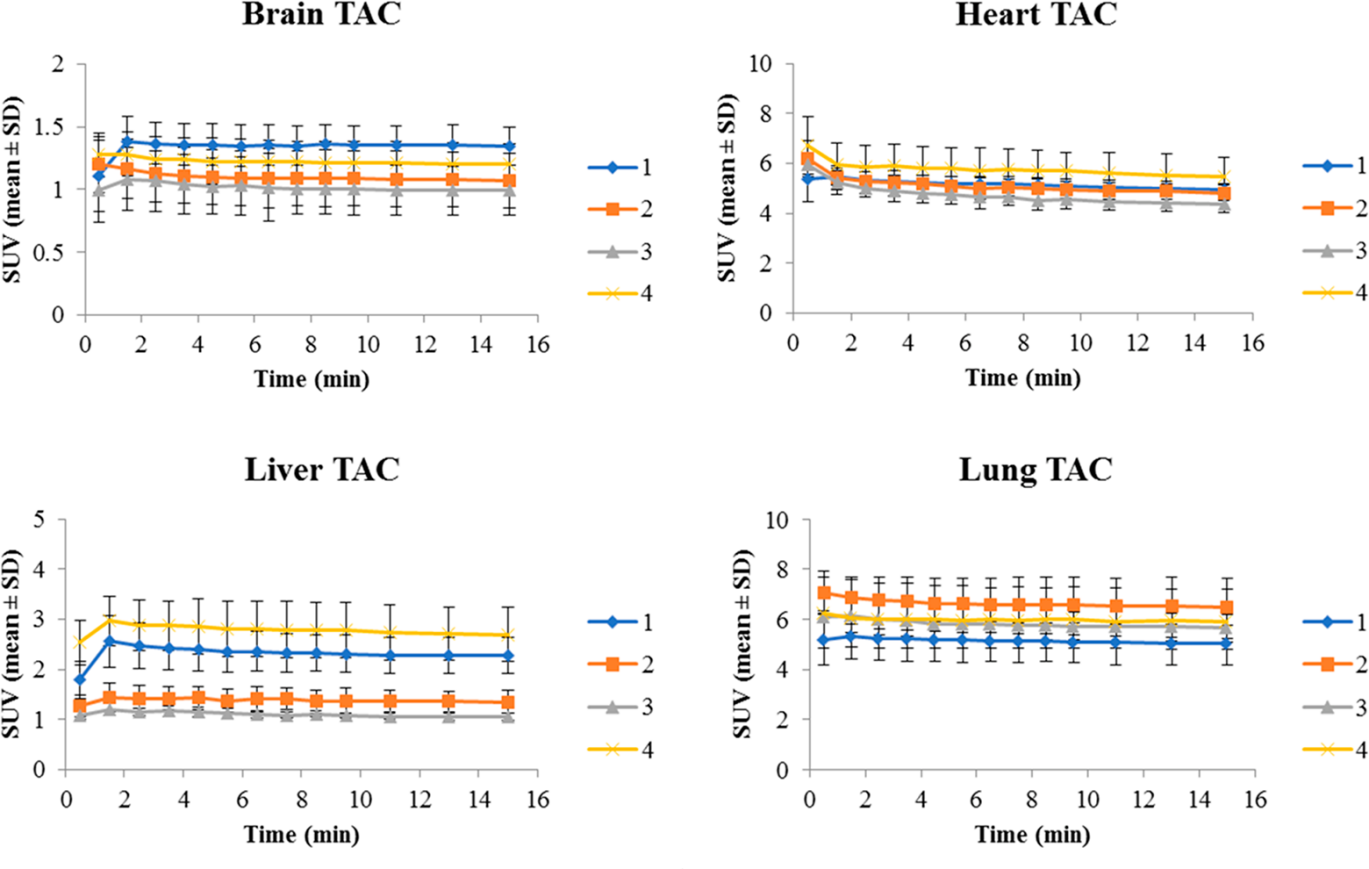
Time-activity curves (TACs) for brain, heart, liver, and lung generated from the microPET/CT data of the [^18^F]VX analog (**1**) blue, [^18^F]sarin analog (**2**) orange, [^11^C]VX analog (**3**) gray, and [^11^C]sarin analog (**4**) yellow. The standard uptake values (SUV) per time point are represented as mean ± SD (*n* ≥ 3).

The sagittal and coronal views from respective PET/CT scans for the four tracers are shown in Figure 2. Corroborating the biodistribution data, modest brain uptake was observed, while greater uptake was seen in the lung, heart, and liver. Brain, heart, liver and lung time-activity curves (TACs) generated from the PET/CT data are shown in Figure 3. An influx of tissue activity was seen over the first 2 min with retention of radioactivity over 15 min. Uptake was rapid in all regions of interest with no significant elimination (washout) observed over the imaging time course. The relative SUV levels in the heart, lung, liver, and brain were similar to the uptake in the biodistribution study, found as lung > heart > liver > brain. While no significant difference was seen in the SUV values among the analog tracers in brain, heart, and liver, which were similar to respective biodistribution profiles; the lung imaging TACs did not demonstrate the same significant differences between the VX and sarin analogs observed in the biodistribution profiles. Although there is no clear reason for the diminished differences between VX and Sarin uptake seen in the lung image analysis, the ∼10–20 fold higher injected radioactivity or interaction with other esterases in the lung tissue may have been contributing factors. This circumstance would not interfere with the assessment of reactivating agents as changes in the brain, blood and clearance of the agents were the key measures for this assessment. The brain TACs did match the biodistribution profiles, where [^18^F]VX tracer was found with the highest amount of radioactivity in brain.

In conjunction with the PET imaging, arterial blood samples were acquired to assess the blood and metabolism profiles of the analog tracers. In order to stabilize the blood samples prior to processing and prevent further interactions between blood proteins and parent tracer analogs and their metabolites, citric acid was used to acidify the sample and neutralize the residual sodium heparin that may accelerate the breakdown the analogs (pH of the sample was not measured). Red blood cells (RBCs) were removed by centrifugation and the proteins were precipitated and separated from the plasma/acetonitrile. The plasma/acetonitrile was analyzed by HPLC to determine the amount of parent tracer in this fraction. All fractions were counted and the percent of total counts for each fraction at time points from 0.5 to 30 min are given in Table 2. As a control, a sample of the formulated analog tracer was subjected to the same blood processing protocol. Greater than 99% of parent tracer survived the citric acid and CH_3_CN processing protocol.

**Table 2:** Distribution of Fluorine-18 and Carbon-11 Analogs in Fractions from the Arterial Blood Samples at 0.5, 1, 5, 10, and 30 min Given As % of Total Counts Represented As the Mean ± SD (*n* ≥ 3)

	time (min)					
	tracer	0.5	1	5	10	30
protein fraction	**1**	71.40 ± 3.40%	70.80 ± 5.00%	77.70 ± 9.20%	79.50 ± 5.50%	80.90 ± 8.00%
	**2**	69.05 ± 4.74%	74.85 ± 6.64%	71.35 ± 15.87%	77.67 ± 4.69%	76.57 ± 4.05%
	**3**	67.07 ± 10.92%	68.70 ± 9.14%	72.30 ± 2.70%	79.88 ± 4.97%	77.25 ± 7.43%
	**4**	84.14 ± 6.65%	82.80 ± 6.35%	88.17 ± 1.96%	86.63 ± 3.59%	90.78 ± 2.74%
red blood cells	**1**	17.40 ± 3.40%	19.20 ± 7.50%	16.40 ± 9.20%	14.90 ± 5.70%	14.90 ± 7.60%
	**2**	22.18 ± 4.14%	15.38 ± 4.83%	20.30 ± 12.47%	16.43 ± 3.10%	18.90 ± 2.46%
	**3**	21.06 ± 11.66%	19.27 ± 10.94%	20.24 ± 2.18%	14.39 ± 4.54%	19.01 ± 7.19%
	**4**	10.48 ± 6.74%	13.47 ± 6.82%	9.45 ± 2.21%	11.55 ± 3.51%	7.58 ± 2.78%
parent tracer	**1**	0.30 ± 0.10%	0.20 ± 0.20%	0.20 ± 0.01%	0.20 ± 0.10%	0.20 ± 0.10%
	**2**	0.49 ± 0.21%	0.36 ± 0.03%	0.18 ± 0.01%	0.11 ± 0.05%	0.07 ± 0.04%
	**3**	0.20 ± 0.22%	0.19 ± 0.18%	0.17 ± 0.15%	0.14 ± 0.05%	0.12 ± 0.01%
	**4**	0.04 ± 0.02%	0.04 ± 0.01%	0.05 ± 0.01%	0.05 ± 0.01%	0.10 ± 0.06%
metabolized tracer	**1**	10.90 ± 0.40%	9.80 ± 3.50%	5.70 ± 0.50%	5.50 ± 0.70%	4.00 ± 0.40%
	**2**	8.28 ± 7.19%	9.41 ± 1.84%	8.17 ± 3.45%	5.80 ± 2.10%	4.46 ± 1.58%
	**3**	11.69 ± 3.11%	11.84 ± 3.00%	7.30 ± 1.19%	5.58 ± 0.77%	3.61 ± 0.70%
	**4**	5.34 ± 0.43%	3.69 ± 0.64%	2.32 ± 0.26%	1.77 ± 0.14%	1.54 ± 0.38%

The majority (88–98%) of the total blood activity for all analog tracers over the first 30 min was associated with the protein fraction (67–90%) and the red blood cells (7.6–22%). The interaction of the tracers and/or metabolites with the RBCs and proteins was corroborated by the elevated blood activity seen in the biodistribution relative to the other tissues sampled, with the exception of the lung tissues. There was no elimination of the activity in the blood over the first 30 min post injection. The identity of the activity associated with these components was not determined so the total parent tracer pool may not be determined. The majority (95–99%) of the activity that remained in the plasma was found in the metabolite fraction. These water-soluble metabolites were not identified but likely include radiolabeled *O*-ethyl/*O*-fluoroethyl-methylphosphonic acid, 2-*O*-propyl/2-*O*-1-fluoropropyl-methylphosphonic acid and/or the radiolabeled alcohols (ethanol and ispropanol).^28^ The hydrolyzed *O*-[^18^F]fluoroethyl-methylphosphonic acid was prepared and injected into rats to show that the labeled acid is not taken up in the brain (results under review in submitted manuscript). All of the analogs rapidly interact with the RBCs and plasma proteins and the plasma soluble activity was largely associated with polar metabolites as early as 0.5 min. All of the analog tracers had similar blood profiles.

**Figure 4:**
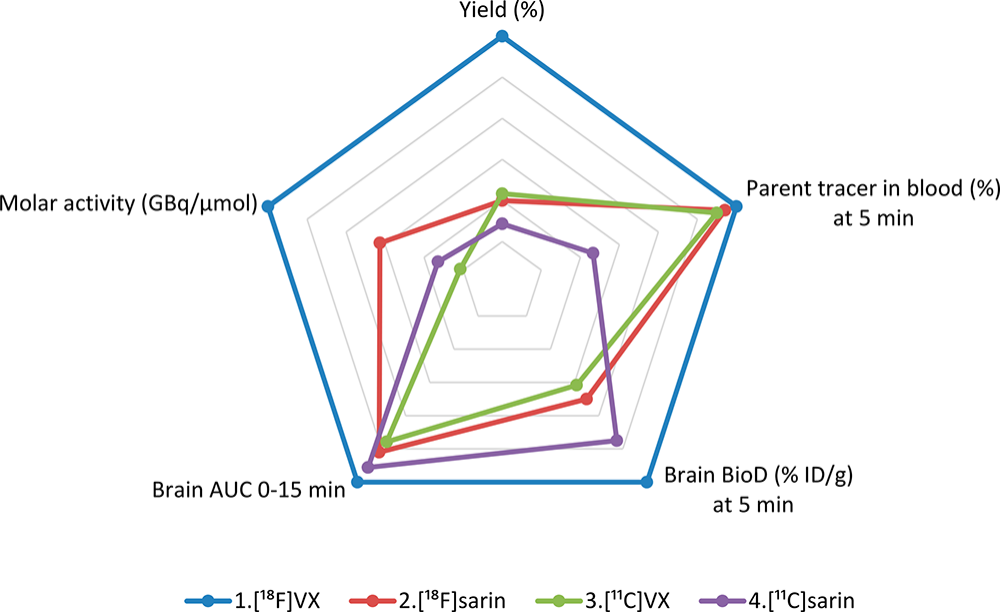
Radar chart of the multivariate analysis of the key comparator metric values for the preparation and in vivo and ex vivo evaluations of the four radiolabeled OP analog tracers **1**–**4**. The relative values for each parameter are shown where the outermost ring (blue) represents relative value = 1 and the innermost ring (gray) represents relative value of 0. AUC is area under the curve and is an integration of the respective PET imaging TAC from 0 to 15 min.

Multivariate analysis was applied to these data to identify the tracer with the more optimal production and in vivo properties; including, synthesis parameters, tracer stability, in vivo radioactivity lifetime, and CNS radioactivity levels. Quantifying changes in brain uptake and interaction with blood components are key measures for OP exposure and prospective reactivation studies. The radiochemical yield and molar activity along with the brain uptake (%ID/g) at 5 min, brain area under the curve (integration of the respective TACs) over the first 15 min after injection and the percent of parent tracer in the blood at 5 min were plotted as a radar chart (Figure 4). The respective variable values on the radar chart were calculated relative to the maximal value for each variable set to a value of 1. Based on all of the data collected and analyzed for the four labeled OP analog tracers **1**-**4**, [^18^F]VX analog, **1**, exhibited the highest values for each of the five variable metrics assessed. Additionally, the longer 110 min half-life of the fluorine-18 was also preferred over the relatively short half-life of 20 min carbon-11, and the higher molar activities of fluorine-18 resulted in a minimal injected tracer mass. Further, the half-life of the fluorine-18 tracers provided a longer window for experimental studies. Measuring the tracer interaction with AChE in the periphery and the CNS were important measures of the OP activity in vivo. Using microPET/CT imaging allowed the study of longitudinal changes post tracer injection and is considered an asset for future oxime reactivation studies in the same animal. Taken together, the outcomes from the in vivo and ex vivo studies performed with OP analog tracers **1**-**4** have established the initial performance qualities for these radiolabeled analog tracers in normal rats that may be used as benchmarks enabling future studies that evaluate the effects of nonradioactive OP exposures, outcomes of current standard of care therapeutic interventions and new pharmacological paradigms.

## Conclusion

In this study, we compared VX (**1** and **3**) and sarin (**2** and **4**) analog tracer ex vivo biodistribution and in vivo distribution profiles concurrently with the blood profiling in Sprague–Dawley rats. There was rapid and sustained uptake of radioactivity in the brain, lung, liver, heart and kidneys over the first 30 min post tracer injections. High serum protein and RBC binding was observed for all of the tracers with <5% parent tracer activity in the plasma fraction. While no statistically significant difference was observed for brain uptake between the four tracers found, the PET imaging brain TAC values and ex vivo biodistribution profiles showed slightly higher mean uptake for the [^18^F]VX analog (**1**) over the other analog tracers (**2**, **3**, and **4**). Considering the five variable metric values as a result of the tracer production and select in vivo and ex vivo profiles, multivariate analysis revealed that the [^18^F]VX tracer **1** was found with the more optimal experimental characteristics. Thus, the [^18^F]VX analog tracer **1** was thought to be suitable for use in future evaluations of OP exposure paradigms and OP-adduct reactivation studies.
